# Prognostic value of metabolic dysfunction-associated steatotic liver disease over coronary computed tomography angiography findings: comparison with no-alcoholic fatty liver disease

**DOI:** 10.1186/s12933-024-02268-1

**Published:** 2024-05-10

**Authors:** Takahiro Nishihara, Toru Miyoshi, Mitsutaka Nakashima, Takashi Miki, Hironobu Toda, Masatoki Yoshida, Keishi Ichikawa, Kazuhiro Osawa, Shinsuke Yuasa

**Affiliations:** 1 Department of Cardiovascular Medicine, Faculty of Medicine, Dentistry and Pharmaceutical Sciences, Okayama University, 2-5-1 Shikata-cho, Kita-ku, 700-8558 Okayama, Okayama Japan; 2 Department of General Internal Medicine 3, Kawasaki Medical School General Medicine Centre, Okayama, Japan

**Keywords:** Metabolic dysfunction-associated fatty liver disease, Coronary computed tomography angiography, High-risk plaque, Obstructive stenosis

## Abstract

**Background:**

Metabolic dysfunction-associated steatotic liver disease (MASLD) is the proposed name change for non-alcoholic fatty liver disease (NAFLD). This study aimed to investigate the association of cardiovascular disease risk with MASLD and NAFLD in patients who underwent clinically indicated coronary computed tomography angiography (CCTA).

**Methods:**

This retrospective study included 2289 patients (60% men; mean age: 68 years) with no history of coronary artery disease who underwent CCTA. The steatotic liver was defined as a hepatic-to-spleen attenuation ratio of < 1.0 on CT just before CCTA. MASLD is defined as the presence of hepatic steatosis along with at least one of the five cardiometabolic risk factors. Adverse CCTA findings were defined as obstructive and/or high-risk plaques. Major adverse cardiac events (MACE) encompassed composite coronary events, including cardiovascular death, acute coronary syndrome, and late coronary revascularization.

**Results:**

MASLD and NAFLD were identified in 415 (18%) and 368 (16%) patients, respectively. Adverse CCTA findings were observed in 40% and 38% of the patients with MASLD and with NAFLD, respectively. Adverse CCTA findings were significantly associated with MASLD (*p* = 0.007) but not NAFLD (*p* = 0.253). During a median follow-up of 4.4 years, 102 (4.4%) MACE were observed. MASLD was significantly associated with MACE (hazard ratio 1.82, 95% CI 1.18–2.83, *p* = 0.007), while its association with NAFLD was not significant (*p* = 0.070). By incorporating MASLD into a prediction model of MACE, including the risk score and adverse CCTA findings, global chi-squared values significantly increased from 87.0 to 94.1 (*p* = 0.008).

**Conclusions:**

Patients with MASLD are likely to have a higher risk of cardiovascular disease than those with NAFLD. Concurrent assessment of MASLD during CCTA improves the identification of patients at a higher risk of cardiovascular disease among those with clinically indicated CCTA.

**Supplementary Information:**

The online version contains supplementary material available at 10.1186/s12933-024-02268-1.

## Background

Non-alcoholic fatty liver disease (NAFLD) is a growing public health concern, with an increasing global prevalence of 30% [1]. It is closely associated with obesity and type 2 diabetes [2]. NAFLD is generally considered a hepatic manifestation of metabolic syndrome [3]. Previous studies have demonstrated that NALFD is a significant predictor of cardiovascular disease (CVD) events [4, 5]. International experts have recently published a consensus statement on new fatty liver disease nomenclature, “steatotic liver disease” (SLD) [6]. SLD is classified as metabolic dysfunction-associated SLD (MASLD), MASLD with increased alcohol intake, alcohol-related liver disease, SLD with a specific etiology, and cryptogenic SLD. MASLD is defined as the presence of hepatic steatosis along with at least one of the five cardiometabolic risk factors that correspond to the components of metabolic syndrome [6].

Coronary computed tomography angiography (CCTA) has been established as an accurate diagnostic tool for assessing obstructive and nonobstructive plaque characteristics [7]. Numerous studies have demonstrated the prognostic value of the presence of adverse CCTA findings, defined as obstructive or high-risk plaques, in patients with suspected coronary artery disease (CAD) [8–10]. The usefulness of computed tomography (CT) as a measure of SLD has also been reported [11]. Our previous research has demonstrated that NAFLD on nonenhanced CT is significantly associated with the presence of high-risk plaques on CCTA and future CVD events in patients with suspected CAD [4].

The updated diagnostic criteria for MASLD require validation regarding the prediction of CVD risks. This study aimed to clarify additional risk stratification benefits of MASLD or NAFLD concurrently assessed during CCTA in patients with suspected stable CAD in a large cohort.

## Methods

### Study population

**Fig. 1:**
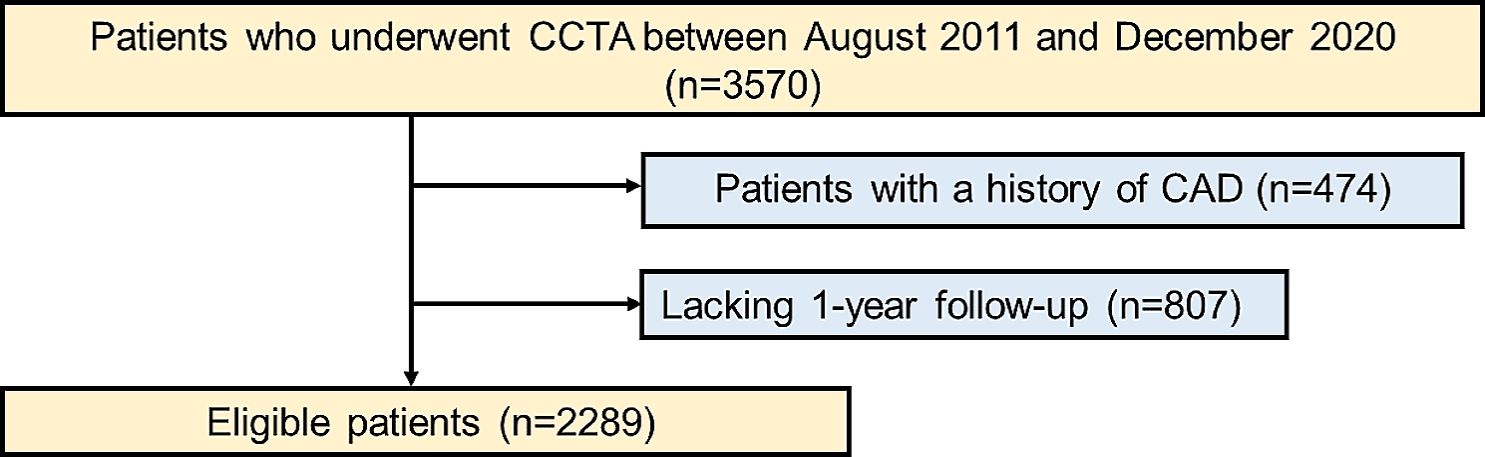
A flow diagram of the study. CAD, coronary artery disease; CCTA, coronary computed tomography angiography

This was a retrospective, single-center cohort study performed at Okayama University Hospital, Japan. Figure 1 shows a flow diagram of the study design. This study enrolled 3570 Japanese outpatients who underwent CCTA between August 2011 and December 2020. Patients with a history of CAD and < 1 year follow-up were excluded. Finally, 2289 patients were included in this study. The study protocol was approved by the Institutional Review Board of Okayama University Hospital, and the study was compliant with the Declaration of Helsinki. Notably, the requirement for informed consent was waived due to the retrospective nature of this study.

### Assessment of risk factors

Detailed definitions of risk factors have been described previously [12]. Patients underwent assessments of height, weight, smoking and alcohol history, and other medical histories through physical examination and medical records. Laboratory values, including triglyceride, low-density lipoprotein cholesterol (LDL-C), high-density lipoprotein cholesterol (HDL-C), and hemoglobin A1c levels, were analyzed at the central laboratory of our hospital. Small dense LDL-C levels were calculated using equations reported by Maureen et al. [13]. We calculated that small dense LDL-C = LDL-C– (1.43 × LDL-C– (0.14 × (ln (triglyceride)×LDL-C))-8.99) [13]. The Hisayama risk score (HRS) was used to classify the study population into low- (< 2%), intermediate- (2–10%), and high-risk (> 10%) groups based on the 10-year atherosclerotic CVD risk [14].

### Computed tomography assessment of hepatic steatosis

CT scans were performed using a 128-slice CT scanner (SOMATOM Definition Flash; Siemens Medical Solutions, Erlangen, Germany) as previously described [15]. An abdominal non-contrast CT scan was conducted immediately before the cardiac scan on the same day, as previously described [16]. The scan range was 20 cm, and the other scan parameters were 120 kVp, 250 mAs, and 5-mm slice thickness. We used a method for assessing steatotic livers consistent with that of previous reports of the Multi-Ethnic Study of Atherosclerosis [17]. Hepatic and splenic Hounsfield attenuations were measured using the mean Hounsfield unit (HU) in the maximum circular regions of interest (at least 1 cm^2^) from the two right liver lobes (anteroposterior dimension) and the spleen. The hepatic-to-splenic attenuation ratio was calculated, and a hepatic-to-spleen attenuation ratio of < 1.0 was defined as a positive diagnosis of steatotic liver [11, 17].

### Diagnoses of NAFLD and MASLD

MASLD was defined based on the evidence of steatotic liver with the presence of 1 or more of the following five metabolic conditions: (i) body mass index ≥ 23 kg/m^2^, waist circumference > 94 cm for males and > 80 cm for females or ethnicity adjusted; (ii) fasting serum glucose ≥ 100 mg/dL, 2-hour post-load glucose levels ≥ 140 mg/dL, or hemoglobin A1c ≥ 5.7%, type 2 diabetes, or treatment for type 2 diabetes; (iii) blood pressure ≥ 130/85 mmHg or specific antihypertensive drug treatment; (iv) plasma triglyceride ≥ 150 mg/dL or lipid-lowering treatment; and (v) plasma HDL-C ≤ 40 mg/dL for males and ≤ 50 mg/dL for females or lipid-lowering treatment [6].

NAFLD was defined as the presence of hepatic steatosis without heavy alcohol consumption (ethanol intake > 30 g/day in men and > 20 g/day in women), other coexisting liver diseases such as hepatitis B or C infections, or the use of medications associated with secondary NAFLD (corticosteroids and amiodarone) [18].

### Acquisition of CCTA and analyses

Coronary CTA images were obtained as described previously [15]. The acquired data were transferred to a workstation (AZE Virtual Place; Canon Medical Systems Corporation, Otawara, Japan) and reconstructed with a slice thickness of 0.625 mm. During CCTA analysis, we evaluated the degree of stenosis and plaque characteristics in segments with a diameter > 2 mm in accordance with the Society of Cardiovascular Computed Tomography [19]. Plaques were categorized as “calcified” (HU > 130), “non-calcified” (HU < 130), or “low-density” (HU < 50) [15]. Moreover, we defined high-risk plaque (HRP) features (positive remodeling; a remodeling index > 1.1, spotty calcification; a calcium burden length < 1.5, and width less than two-thirds of the vessel diameter, low-density plaque; HU < 30) as previously described [20]. The presence of *≥* 2 features was defined as HRP. Significant stenosis was defined as a luminal narrowing *≥* 50%. Adverse CCTA findings were defined as the presence of significant stenosis and/or HRP. Two experienced cardiovascular imagers (T.N. and T.M.) who were blinded to the clinical data analyzed the CCTA images.

### Outcome data

Clinical follow-up was performed by reviewing medical records or telephone interviews. Major adverse cardiac events (MACE) were defined as the composite of cardiovascular death, nonfatal myocardial infarction, and late coronary revascularization. Each outcome was reviewed by clinical event review members (M.N. and T.M.) who were blinded to the CT results according to the relevant criteria. Details of the event definitions are provided in the Additional file. Cardiac death was defined as death due to any of the following causes: acute coronary syndrome (ACS), heart failure, arrhythmic death, or unclear causes of death in which a cardiac origin could not be excluded. ACS includes myocardial infarction and unstable angina. Late coronary revascularization was defined as planned percutaneous coronary intervention or coronary artery bypass grafting due to stable CAD with a new positive functional test for ischemia > 90 days after coronary CTA. MACE occurrence in patients with revascularization scheduled within 90 days on indexed coronary CT findings was excluded to eliminate confounding factors, and these patients were censored at the time of the first revascularization.

### Statistical analysis

Continuous variables are expressed as mean ± standard deviation or median with interquartile range. Categorical variables are presented as counts (n) and percentages (%). Continuous variables were compared using the paired Student’s *t*-test or Mann–Whitney U-test, whereas categorical variables were compared using chi-squared (*χ*^2^) analysis or Fisher’s exact test. Cumulative survival estimates were calculated using the Kaplan–Meier method and compared using the log-rank test. The Kaplan–Meier method was applied after categorizing the participants into four groups based on the presence of MASLD or NAFLD and adverse CCTA findings. We performed univariate and multivariate logistic regression analysis to evaluate determinants of adverse CCTA findings, and the results are presented as odds ratios (ORs) with 95% confidence intervals (CIs). The multivariate logistic regression model included age, sex, chronic kidney disease (CKD), current smoking status, and low-density LDL-C. Statin use was also included as a variable. To avoid overlap with the MASLD definition, body mass index, hypertension, dyslipidemia and type 2 diabetes were excluded. To investigate the association of MASLD and NAFLD with MACE, we conducted univariate and multivariate Cox regression analyses, and the results are presented as hazard ratios (HRs) with 95% CIs. The multivariate Cox regression model included the same variables as the multivariate logistic regression model and adverse CT findings. The Hisayama risk score was excluded to avoid overlap with factors in the multivariate model. In the Cox regression model, time was defined as the duration from the baseline to the occurrence of an event or the end of the follow-up period. Furthermore, we assessed the additional predictive value of the presence of MASLD and NAFLD in comparison to adverse CCTA findings for predicting MACE using the global *χ*^2^ test. A *p*-value < 0.05 was considered statistically significant. All statistical analyses were performed using SPSS software (version 29; IBM Corp., Armonk, NY, USA) and the R statistical package (version 4.1.1; R Foundation for Statistical Computing, Vienna, Austria).

## Results

### Patient characteristics

**Fig. 2:**
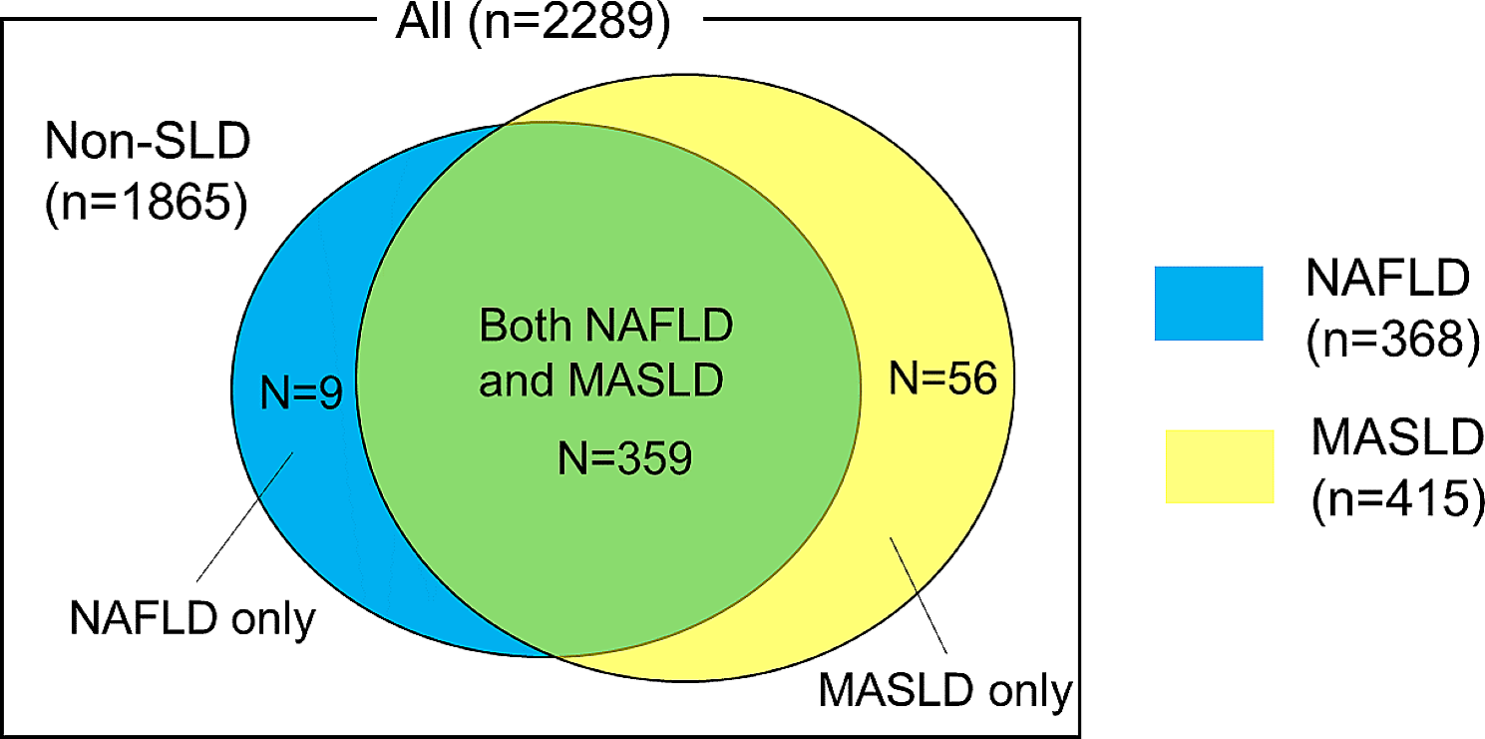
Prevalence of MAFLD and NAFLD. MASLD, metabolic dysfunction-associated steatotic liver disease; NAFLD, non-alcoholic fatty liver disease; SLD, steatotic liver disease

The mean age of the study population was 68 years, and 1371 (60%) patients were men. Among 2289 patients included in the study, 415 (18%) and 368 (16%) were diagnosed with MASLD and NAFLD, respectively. Using the new definition, 56 (2.4%) patients previously not classified as having NAFLD were newly identified as having MASLD (MASLD only) (Fig. 2). Conversely, 9 (0.4%) patients who had been previously classified as having NAFLD did not meet the MASLD criteria (NAFLD only). The remaining 359 (15.6%) patients met both MASLD and NAFLD criteria.

Baseline characteristics of the patients were comparable between those with MASLD and those with NAFLD (Table 1). Patients with MASLD or NAFLD were more likely to be young, male, and to have a higher body mass index, hypertension, dyslipidemia, type 2 diabetes, and CKD than those without MASLD or NAFLD. Additionally, lipid profiles (triglyceride, total cholesterol, HDL-C, LDL-C, small dense LDL-C), AST, and ALT in patients with MASLD or NAFLD were worse than those in patients without MASLD or NAFLD. However, Patients with MASLD were more likely to have elevated HRS compared with those with NAFLD.

**Table 1:** Patient characteristics according to the presence of NAFLD and MASLD

	All (*n* = 2289)	Non-NAFLD ( n = 1921)	NAFLD (*n* = 368)	*p*-value	Non-MASLD ( n = 1874)	MASLD (*n* = 415)	*p*-value
Age, years	68 (57, 74)	68 (58, 75)	62 (52, 70)	< 0.001	68 (58, 75)	63 (53, 71)	< 0.001
Male sex, n (%)	1371 (60)	1132 (59)	239 (65)	0.031	1089 (58)	282 (68)	< 0.001
Body mass index, kg/m^2^	23 (21, 26)	23 (21, 25)	26 (24, 29)	< 0.001	23 (21, 25)	26 (24, 29)	< 0.001
Hypertension, n (%)	1338 (59)	1081 (56)	257 (70)	< 0.001	1042 (56)	296 (71)	< 0.001
Dyslipidemia, n (%)	1012 (44)	816 (43)	196 (53)	< 0.001	790 (42)	222 (54)	< 0.001
Type 2 diabetes, n (%)	638 (28)	476 (25)	162 (44)	< 0.001	447 (24)	191 (46)	< 0.001
Chronic kidney disease, n (%)	683 (30)	595 (31)	88 (24)	0.007	584 (31)	99 (24)	0.003
Current smoker, n (%)	392 (17)	316 (16)	76 (21)	0.050	301 (16)	91 (22)	0.004
Beta blocker, n (%)	633 (28)	516 (27)	117 (32)	0.053	501 (27)	132 (32)	0.037
Calcium channel blocker, n (%)	795 (35)	652 (34)	143 (39)	0.069	629 (34)	166 (40)	0.013
ACE-I or ARB, n (%)	841 (37)	684 (36)	157 (43)	0.010	661 (35)	180 (43)	0.002
Statin, n (%)	680 (30)	552 (29)	128 (35)	0.020	539 (29)	141 (34)	0.035
Oral antihyperglycemic drugs, n (%)	364 (16)	258 (13)	106 (29)	< 0.001	238 (13)	126 (30)	< 0.001
AST, IU/L	21 (17, 27)	21 (17, 26)	24 (19, 32)	< 0.001	20 (17, 25)	25 (20, 32)	< 0.001
ALT, IU/L	18 (13, 25)	17 (12, 23)	28 (19, 39)	< 0.001	16 (12, 23)	28 (19, 40)	< 0.001
eGFR, ml/min/1.73 m^2^	68 (57, 80)	67 (57, 80)	69 (60, 81)	0.009	67 (57, 80)	69 (60, 81)	0.004
Hemoglobin A1c, %	5.9 (5.6, 6.4)	5.9 (5.6, 6.3)	6.2 (5.8, 6.9)	< 0.001	5.9 (5.6, 6.3)	6.2 (5.8, 7.0)	< 0.001
Triglyceride, mg/dl	112 (81, 164)	107 (78, 157)	137 (102, 202)	< 0.001	105 (78, 154)	146 (106, 221)	< 0.001
Total cholesterol, mg/dl	190 ± 38	189 ± 39	192 ± 37	0.278	189 ± 39	193 ± 38	0.054
HDL cholesterol, mg/dl	57 (47, 69)	59 (48, 70)	49.0 (43, 59)	< 0.001	59 (48, 71)	50 (42, 59)	< 0.001
LDL cholesterol, mg/dl	109 (91, 133)	108 (90, 132)	113 (95, 137)	0.012	108 (89, 132)	114 (96, 137)	0.001
Small-dense LDL cholesterol, mg/dl	34 (27, 43)	33 (26, 42)	38 (31, 47)	< 0.001	33 (26, 42)	39 (32, 49)	< 0.001
Hisayama risk score, n (%)							
Low-risk	535 (23)	444 (23)	91 (25)	0.013	444 (24)	91 (22)	0.134
Intermediate-risk	1180 (52)	973 (51)	207 (56)		948 (51)	232 (56)	
High-risk	574 (25)	504 (26)	70 (19)		482 (26)	92 (22)	
Adverse CTA findings, n (%)	811 (35)	671 (35)	140 (38)	0.253	646 (35)	165 (40)	0.042
High-risk plaque, n (%)	474 (21)	379 (20)	95 (26)	0.008	364 (19)	110 (27)	0.001
Obstructive plaque, n (%)	623 (27)	518 (27)	105 (29)	0.536	497 (27)	126 (30)	0.112

Data are presented as mean ± standard deviation, n (%), or median [25th–75th percentile]. Adverse CTA findings were defined as obstructive and/or high-risk plaques

ACE-Is, angiotensin–converting–enzyme inhibitors; ALT, alanine aminotransferase; ARBs, angiotensin receptor blockers; AST, aspartate aminotransferase; CCB, calcium channel blocker; CTA, computed tomography angiography; eGFR, estimated glomerular filtration rate; HDL, high-density lipoprotein; LDL, low-density lipoprotein

### Plaque characteristics of MASLD and NAFLD

Plaque characteristics were compared between patients with and without MASLD and between patients with and without NAFLD. As shown in Table 1, patients with both MASLD and NAFLD had a significantly higher prevalence of HRP than those without MASLD and NAFLD (*p* = 0.001 and *p* = 0.008, respectively). However, a significant difference in the prevalence of adverse CT findings was observed between patients with and without MASLD rather than between patients with and without NAFLD (*p* = 0.042 and *p* = 0.253, respectively).

In Table 2, logistic regression analysis was performed to evaluate determinants of adverse CCTA findings. In the univariate logistic regression analysis, adverse CCTA findings were associated with MASLD (*p* = 0.039) rather than NAFLD (*p* = 0.253). Moreover, in the multivariable logistic regression analysis, including variables (age, sex, CKD, current smoking status, statin use, and small dense LDL-C), the association between adverse CCTA findings and MASLD remained significant (*p* = 0.042).

**Table 2:** Factors associated with adverse CT findings

	Univariate		Multivariate*	
	OR (95% CI)	*P* value	OR (95% CI)	*P* value
Age	1.05 (1.04–1.06)	< 0.001	1.05 (1.04–1.06)	< 0.001
Male sex	2.36 (1.96–2.84)	< 0.001	2.66 (2.12–3.34)	< 0.001
Body mass index	1.04 (1.02–1.06)	< 0.001		
Hypertension	2.61 (2.17–3.14)	< 0.001		
Dyslipidemia	2.15 (1.80–2.56)	< 0.001		
Type 2 diabetes	2.49 (2.06-3.00)	< 0.001		
Chronic kidney disease	1.42 (1.18–1.71)	< 0.001	0.86 (0.68–1.08)	0.185
Current Smoker	1.37 (1.10–1.71)	0.005	1.16 (0.88–1.52)	0.307
Beta blocker	0.97 (0.80–1.17)	0.749		
Calcium channel blocker	1.75 (1.46–2.09)	< 0.001		
ACE-I or ARB	1.93 (1.62–2.30)	< 0.001		
Statin	1.92 (1.60–2.31)	< 0.001	1.96 (1.58–2.43)	< 0.001
Oral antihyperglycemic drugs	2.62 (2.09–3.29)	< 0.001		
Small dense LDL-cholesterol	1.01 (1.00-1.01)	0.070	1.01 (1.00-1.02)	0.044
NAFLD	1.14 (0.91–1.44)	0.253		
MASLD	1.27 (1.01–1.59)	0.039	1.31 (1.01–1.71)	0.042

ACE-Is, angiotensin–converting–enzyme inhibitors; ARBs, angiotensin receptor blockers; CI, confidence interval; OR, odds ratio; LDL, low-density lipoprotein; NAFLD, nonalcoholic fatty liver disease; MASLD, metabolic dysfunction-associated steatotic liver disease

*Multivariate analysis included age, sex, chronic kidney disease, current smoking status, statin use, and MASLD score

### Association of MASLD and NAFLD with MACE

**Fig. 3:**
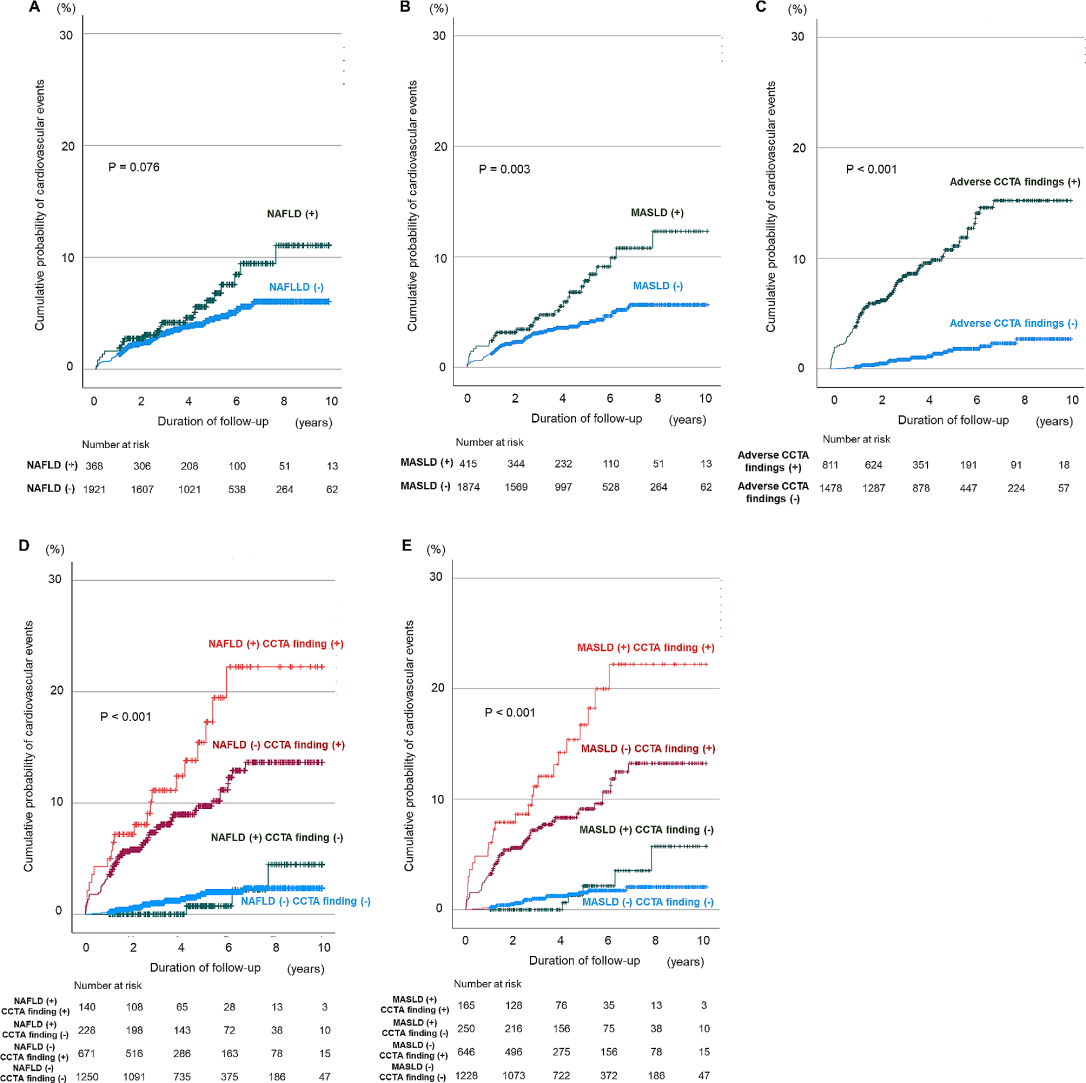
Kaplan–Meier curves stratified according to NAFLD, MASLD, and adverse CCTA findings for MACE. The incidence of MACE during follow-up according to the presence or absence of NAFLD (**A**), the presence or absence of MASLD (**B**), the presence or absence of adverse CCTA findings (**C**), a combination of NAFLD and adverse CCTA findings (**D**), and a combination of MASLD and adverse CCTA findings (**E**) CCTA, coronary computed tomography angiography; MASLD, metabolic dysfunction-associated steatotic liver disease; NAFLD, non-alcoholic fatty liver disease

Overall, 102 CVD events were documented during a median follow-up of 4.4 years. Among these, 28 events occurred in patients with MASLD, comprising 3 cardiovascular deaths, 8 myocardial infarctions, and 17 late revascularizations; and 74 events in patients without MASLD: 13 cardiovascular deaths, 13 myocardial infarctions, and 48 late revascularizations). Furthermore, 23 events were observed in patients with NAFLD as follows: 3 cardiovascular deaths, 7 myocardial infarctions, and 13 late revascularization; and 79 events in patients without NAFLD as follows: 13 cardiovascular deaths, 14 myocardial infarctions, and 52 late revascularization. When all participants were categorized according to the presence of MASLD or NAFLD, Kaplan–Meier curves showed that patients with MASLD had higher event rates than patients without MASLD but not NAFLD (Fig. 3A and B; log-rank test, *p* = 0.003 and *p* = 0.076). When all participants were categorized according to the presence of adverse CCTA findings, the Kaplan–Meier curves showed that patients with adverse CCTA findings had higher event rates than those without adverse CCTA findings in Fig. 3C (log-rank test, *p* < 0.001). When all participants were categorized according to the combination of MASLD or NAFLD and adverse CCTA findings, Kaplan–Meier curves showed that patients with both MASLD or NAFLD and adverse CCTA findings had the highest event rates compared to patients without MASLD or NAFLD and adverse CCTA findings (Fig. 3D and E; log-rank test, *p* < 0.001).

As shown in Table 3, univariate Cox regression analysis showed that MASLD was associated with MACE. Furthermore, in the multivariate Cox regression analysis adjusted for age, sex, CKD, current smoking status, statin use, small dense LDL-C, and adverse CCTA findings, the presence of MASLD was associated with MACE (*p* = 0.008). However, the presence of NAFLD was not significantly associated with MACE (*p* = 0.065).

**Table 3:** The association between NAFLD and MAFLD and adverse cardiovascular events

	Univariate		Multivariate 1		Multivariate 2	
	HR (95% CI)	*p*-value	HR (95% CI)	*p*-value	HR (95% CI)	*p*-value
Age, years	1.03 (1.02–1.05)	< 0.001	1.02 (1.00–1.04)	0.100	1.02 (1.00–1.04)	0.081
Male sex	1.95 (1.26–3.01)	0.003	1.09 (0.67–1.77)	0.740	1.05 (0.64–1.71)	0.859
Body mass index	1.01 (0.97–1.06)	0.581				
Hypertension	1.66 (1.09–2.52)	0.018				
Dyslipidemia	1.71 (1.16–2.53)	0.007				
Diabetes mellitus	1.76 (1.19–2.60)	0.005				
Chronic kidney disease	1.56 (1.05–2.33)	0.028	1.37 (0.88–2.12)	0.161	1.40 (0.90–2.16)	0.135
Current Smoker	2.25 (1.48–3.41)	< 0.001	2.06 (1.29–3.29)	0.003	2.09 (1.31–3.34)	0.002
Beta blocker	1.04 (0.67–1.61)	0.857				
Calcium channel blocker	1.33 (0.90–1.98)	0.154				
ACE-I or ARB	1.23 (0.83–1.82)	0.302				
Statin	1.17 (0.77–1.76)	0.462	0.78 (0.50–1.23)	0.285	0.78 (0.50–1.22)	0.778
Oral antihyperglycemic drugs	2.02 (1.32–3.11)	0.001				
Hisayama risk score	2.18 (1.62–2.93)	< 0.001				
Small dense LDL-cholesterol	1.01 (1.00–1.03)	0.117	1.01 (0.99–1.02)	0.530	1.00 (0.99–1.02)	0.658
Adverse CCTA findings	7.38 (4.64–11.73)	< 0.001	6.00 (3.56–10.09)	< 0.001	5.95 (3.54–10.01)	< 0.001
NAFLD	1.52 (0.95–2.41)	0.078	1.60 (0.97–2.63)	0.065		
MASLD	1.89 (1.24–2.90)	0.003			1.88 (1.18–3.00)	0.008

ACE-Is, angiotensin–converting–enzyme inhibitors; ARBs, angiotensin receptor blockers; LDL, low-density lipoprotein; CI, confidence interval; HR, hazard ratio; NAFLD, nonalcoholic fatty liver disease; MASLD, metabolic dysfunction-associated steatotic liver disease

### Comparison of predictive performances for MACE

**Fig. 4:**
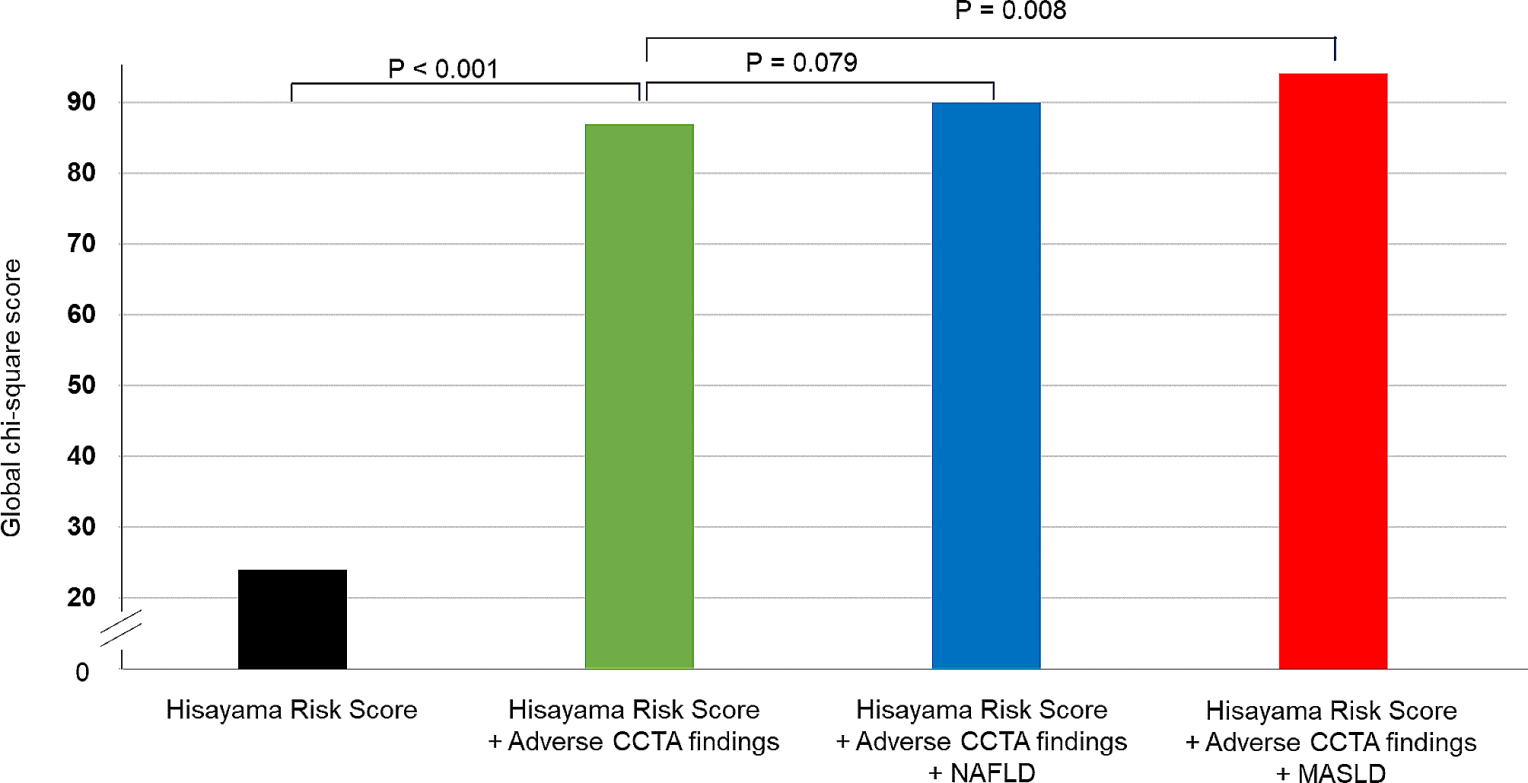
The incremental predictive value of NAFLD or MASLD and adverse CT findings and the HRS. A global χ 2 test was used to evaluate the model fitness through adding NAFLD or MASLD for the prediction of MACE in relation to a model of adverse CCTA finding and the Hisayama risk score. CCTA, coronary computed tomography angiography; HRS, Hisayama risk score; MASLD, metabolic dysfunction-associated steatotic liver disease; NAFLD, non-alcoholic fatty liver disease

Finally, we assessed whether the inclusion of MASLD or NAFLD to adverse CCTA findings and HRS improved the risk stratification for MACE. Figure 4 illustrates the incremental value of adverse CCTA findings and MASLD or NAFLD in predicting MACE. By considering MASLD along with adverse CCTA findings and HRS, the global *χ*^2^ value significantly increased from 87.0 to 94.1 (*p* = 0.008), while not in NAFLD (*p* = 0.079). The net reclassification index achieved by incorporating MASLD to adverse CTA findings and HRS was 0.236 (95% confidence interval 0.056–0.415, *p* = 0.010), while that achieved by adding NAFLD was 0.135 (-0.02 to 0.300, *p* = 0.107).

## Discussion

This study demonstrated that MASLD, which was associated with adverse CCTA findings defined as obstructive stenosis and/or HRP, was associated with a higher risk of MACE than NAFLD. Moreover, the presence of MASLD, concurrently assessed during CCTA, along with adverse CCTA findings, enhanced the risk prediction of MACE in patients with clinically indicated CCTA.

To date, no study has reported an increased risk of CVD events in patients with metabolic dysfunction-associated fatty liver disease (MAFLD) compared to those with NAFLD. Previous studies have shown that the higher the number of metabolic components present in individuals with NAFLD, the higher the risk of mortality, highlighting the important roles of metabolic factors in the natural history of NAFLD [21, 22]. In 2020, a new concept called MAFLD was proposed [23]. MAFLD is diagnosed when liver steatosis is present in individuals who are overweight or obese, have type 2 diabetes, or exhibit at least two metabolic risk abnormalities [23]. Although variance between MASLD and MAFLD is anticipated, several studies have reported that MAFLD predicts the risk of CVD events better than NAFLD [24, 25]. The findings of our study are consistent with the importance of metabolic components in cardiovascular outcomes in patients with SLD. The criteria for MASLD include one or more of five cardiometabolic risk factors, thus enabling the identification of patients at a higher risk of CVD.

NAFLD and CVD both share several common metabolic risk factors such as genetics, systemic inflammation, endothelial dysfunction, hepatic insulin resistance, adipose tissue dysfunction, oxidative stress, and lipid metabolism [26, 27]. Moreover, NAFLD is closely linked with various metabolic conditions, which predispose individuals to an elevated risk of CVD [28, 29]. As a result, the patients with NAFLD have tendency to change the composition of serum lipoproteins like smaller peak diameter and particle size and higher particle concentration of LDL-C [30], which was consistent with the result in the present study.

This study revealed that MASLD was more useful than NAFLD in predicting CVD events. There are several possible explanations for these results. First, adverse CCTA findings, including high-risk plaques and significant stenosis, were significantly associated with rather than NAFLD. As shown in this study, adverse CCTA findings significantly affected the incidence of CVD events. Moreover, in this study, patients with MASLD were likely to have a greater high-risk group for HRS than those with NAFLD (22% vs. 19%, respectively). HRS is a risk prediction model for the development of atherosclerotic CVD in Japanese adults [14]. The inclusion criteria for MASLD may have facilitated the identification of the high-risk group for CVD more accurately than those for NAFLD.

This study demonstrated that MASLD concurrently assessed during CCTA significantly improved CVD risk stratification. Performing early and accurate MASLD assessments during CVD risk assessment is crucial. In clinical practice, ultrasonography is typically used to diagnose fatty infiltration; however, non-contrast CT is a useful method for diagnosing liver fat with wide generalization [11]. Based on our findings, utilizing this approach in comprehensive CCTA can enhance the risk stratification of CVD.

Currently, there are no approved medical treatments for MASLD. The primary treatment comprises weight loss through lifestyle interventions, similar to the approach used for NAFLD [31, 32]. Diet and exercise have been found to improve histology, with a greater reduction in inflammation and fibrosis [33]. In patients with type 2 diabetes, pioglitazone, glucagon-like peptide-1 receptor agonists, and sodium glucose cotransporter 2 inhibitors are recommended to improve liver fibrosis [34]. Statins improve cardiovascular outcomes in patients with NAFLD in association with improved aminotransferase levels [35, 36]. Pemafibrate therapy improves markers of hepatic inflammation and fibrosis, regardless of body mass index [37]. These drugs may be effective in improving the prognosis of patients with MASLD. Further studies are required to restore the steatotic liver and interrupt inflammatory and fibrogenic processes.

This study has some limitations. First, the study population was comprised solely by Japanese patients and conducted at a single center. The median age in this study was older than previous studies. Therefore, the results cannot be generalized to other ethnic groups and younger age groups. Second, this study had selection bias because it targeted only patients who underwent clinically indicated CCTA. The prevalence of MASLD (approximately 18% diagnosed using abdominal CT among the enrolled patients) was lower than that reported in previous studies. This discrepancy may be attributed to the differences in the study population, as the enrolled patients in this study, who had clinically indicated CCTA, were different from those in other studies, and the steatotic liver was mostly diagnosed using ultrasonography and magnetic resonance imaging in previous studies. Third, CT results alone may not be sufficient to diagnose SLD, and other examinations other than CT, such as ultrasonography and blood biomarkers, were not performed in our study. Fourth, we did not collect information on changes in medication and risk factor control during the follow-up period, potentially influencing the risk estimates for MASLD. Fifth, our study outlined the feasibility of the simultaneous examination of SLD during CCTA in assessing the risk of cardiovascular events. CCTA is not recommended for a screening of asymptomatic patients. Finally, this was a retrospective observational study. We cannot define a cause-and-effect relationship between MASLD and CVD.

## Conclusion

This study demonstrated that the presence of MASLD is significantly associated with MACE and that patients with MASLD may have a higher risk of MACE than those with NAFLD. Moreover, MASLD improved the predictive ability of MACE in addition to adverse CCTA findings in patients who underwent clinically indicated CCTA. Concurrently evaluating MASLD during comprehensive CCTA is effective in identifying patients at a higher risk of CVD events.

### Supplementary Information

Supplementary material 1

## Data Availability

The datasets used and/or analysed during the current study are available from the corresponding author on reasonable request.

## Abbreviations

CAD: coronary artery disease
CCTA: coronary computed tomography angiography
CKD: chronic kidney disease
CVD: cardiovascular disease
HDL-C: high-density lipoprotein cholesterol
HR: hazard ratio
HRP: high-risk plaque
HRS: Hisayama risk score
HU: Hounsfield unit
MACE: major adverse cardiac events
MASLD: metabolic dysfunction-associated SLD
LDL-C: low-density lipoprotein cholesterol
MAFLD: metabolic dysfunction-associated fatty liver disease
NAFLD: non-alcoholic fatty liver disease
SLD: steatotic liver disease
