# Supplementary material for: Prognostic value of metabolic dysfunction-associated steatotic liver disease over coronary computed tomography angiography findings: comparison with no-alcoholic fatty liver disease
Source: Cardiovasc Diabetol. 2024 May 10;23:167. doi: 10.1186/s12933-024-02268-1 (PMC11088086; doi:10.1186/s12933-024-02268-1)
Supplement: Supplementary file 1 — Supplementary material 1 [file 12933_2024_2268_MOESM1_ESM.docx]

**−Supplemental Material−**

**Definition of major adverse cardiac events**

**Supplemental Table I**

**Definition of major adverse cardiac events**

Major adverse cardiac events (MACE) include cardiovascular death, acute coronary syndrome, and late coronary revascularization.

**• Cardiovascular death**

The cause of death was determined by the principal condition that caused the death, not the immediate mode of death. Clinical Events Committee members reviewed all available information and used their clinical expertise to adjudicate the cause of death. All deaths not attributed to the categories of CV death and not attributed to a non-CV cause were presumed CV deaths. Death certificates or summary, if possible, were provided for all patients who died, including date and details surrounding death. However, if a death certificate was the only information available for review besides the patient profile in the clinical trial database, the Clinical Events Committee may have decided not to use this information as cause of death if another aetiology appeared more plausible. The following definitions were used for the adjudication of fatal cases:

*Sudden cardiac death.*

Death that occurs unexpectedly in a previously stable patient and includes the following deaths:

- Witnessed and instantaneous death without new or worsening symptoms
- Witnessed and instantaneous without new or worsening symptoms
- Witnessed within 60 minutes of the onset of new or worsening cardiac symptoms
- Witnessed and attributed to an identified arrhythmia (e.g., captured on ECG recording or witnessed on a monitor by either a medic or paramedic)
- Subjects unsuccessfully resuscitated from cardiac arrest or successfully resuscitated from cardiac arrest but who die within 24 hours without identification of a non-cardiac aetiology
- Unwitnessed death and there is no conclusive evidence of another, non-CV, cause of death (i.e. presumed CV death)

*Sudden death due to acute myocardial infarction (MI) (MI type 3).*

Sudden death occurring up to 14 days after a documented acute MI (verified either by the diagnostic criteria outlined for acute MI or by autopsy findings showing recent MI or recent coronary thrombus) and where there is no conclusive evidence of another cause of death. If death occurs before biochemical confirmation of myocardial necrosis can be obtained, adjudication should be based on clinical presentation and ECG evidence.

*Death attributable to heart failure or cardiogenic shock.*

Death occurring in the context of clinically worsening symptoms and/or signs of congestive heart failure (CHF) without evidence of another cause of death. New or worsening signs and/or symptoms of CHF include any of the following:

- New or increasing symptoms and/or signs of heart failure requiring the initiation of, or an increase in, treatment directed at heart failure or occurring in a patient already receiving maximal therapy for heart failure
- Heart failure symptoms or signs requiring continuous intravenous therapy or oxygen administration
- Confinement to bed predominantly because of heart failure symptoms
- Pulmonary edema sufficient to cause tachypnea and distress not occurring in the context of an acute MI or as the consequence of an arrhythmia occurring in the absence of worsening heart failure
- Cardiogenic shock not occurring in the context of an acute MI or as the consequence of an arrhythmia occurring in the absence of worsening heart failure

– Cardiogenic shock is defined as systolic blood pressure (SBP) <90 mmHg for more than 1 hour, ack of response to fluid resuscitation and/or heart rate correction, and judged to be secondary to cardiac dysfunction and associated with at least one of the following signs of hypoperfusion:

1. Cool, clammy skin

2. Oliguria (urine output<30 mL/hour)

3. Altered sensorium

4. Cardiac index<2.2L/min/m^2^

Cardiogenic shock can also be defined in the presence of SBP ≥90 mmHg or for a time period <1 hour if the blood pressure measurement or time period is influenced by the presence of positive inotropic or vasopressor agents alone and/or with mechanical support<1 hour. The outcome of cardiogenic shock will be based on the review member’s assessment. This category will include sudden death occurring during an admission for worsening heart failure

*Death due to stroke, cerebrovascular event*

Death occurring up to 30 days after a stroke that is either due to the stroke or caused by complication of the stroke.

*Death due to other CV causes*

Death must be due to a fully documented CV cause not included in the above categories (e.g. dysrhythmia, pulmonary embolism, or CV intervention). Death due to a MI that occurs as a direct consequence of a CV investigation/procedure/ operation will be classified as death due to other CV cause.

**• Acute coronary syndrome**

ACS includes (1) MI and (2) hospitalization for unstable angina.

1. MI (non-fatal)

Myocardial infarction includes type 1 and type 2 myocardial infarction.

*Spontaneous MI (type 1)*

To identify a type 1 MI, patients should demonstrate spontaneous symptoms of myocardial ischemia unprovoked by supply/demand inequity, together with ≥1 of the following criteria:

• Cardiac biomarker elevation: Troponin is the preferred marker for adjudicating the presence of acute MI. At least one value should show a rise and/or fall from the lowest cut-point providing 10% imprecision (typically the upper reference limit for the troponin run per standard of clinical care). Creatine kinase-MB is a secondary choice of marker to troponin; a rise in CK-MB above the local upper reference limit would be consistent with myocardial injury.

• ECG changes consistent with new ischemic changes

– ECG changes indicative of new ischemia (new ST-T changes or new left bundle branch block [LBBB]) or ECG manifestations of acute myocardial ischemia (in the absence of left ventricular hypertrophy [LVH] and LBBB):

– Development of pathological Q waves in the ECG

1. Any Q-wave in leads V2–V3 ≥0.02 seconds or QS complex in leads V2 and V3

2. Q-wave ≥0.03 seconds and ≥0.1 mV deep or QS complex in leads I, II, aVL, aVF, or V4- V6 in any two leads of a contiguous lead grouping (I, aVL, V6; V4-V6; II, III, and aVF)

– ST elevation: New ST elevation at the J-point in two contiguous leads with the cut-off points: ≥0.2 mV in men or ≥0.15 mV in women in leads V2–V3 and/or ≥0.1 mV in other leads

– ST depression and T-wave changes: New horizontal or down-sloping ST depression ≥0.05 mV in two contiguous leads and/or T inversion ≥0.1 mV in two contiguous leads with prominent R-wave or R/S ratio >1

• Imaging evidence of new non-viable myocardium or new wall motion abnormality

*“Demand”-related (type 2) MI*

Patients with type 2 MI should be considered under similar diagnostic criteria as a type 1 MI; however, type 2 MI should be considered present when myocardial ischemia and infarction are consequent to supply/demand inequity, rather than a spontaneous plaque rupture and coronary thrombosis.

1. Hospitalization for Unstable angina

The date of this event will be the day of hospitalization of the patient including any overnight stay at an emergency room or chest pain unit. Unstable angina requiring hospitalization is defined as all of the following:

- No elevation in cardiac biomarkers (cardiac biomarkers negative for myocardial necrosis) according to conventional assays or contemporary sensitive assays
- Clinical presentation: Cardiac symptoms lasting ≥10 minutes and considered to be myocardial ischemia upon final diagnosis with one of the following:

– Rest angina

– New-onset (<2 months) severe angina (Canadian Cardiovascular Society [CCS] grading Scale, or CCS classification system, classification severity ≥III)

–Increasing angina (in intensity, duration, and/or frequency) with an increase in severity of >1 CCS class to CCS class >III

- Angina requiring an unscheduled visit to a healthcare facility and overnight admission

At least one of the following:

– New or worsening ST or T-wave changes by ECG. ECG changes should satisfy the following criteria for acute myocardial ischemia in the absence of LVH and LBBB:

1. ST elevation: New transient (known to be <20minutes) ST elevation at the J-point in two contiguous leads with cut-off points of ≥0.2 mV in men or ≥0.15 mV in women in leads V2–V3 and/or ≥0.1 mV in other leads
2. ST depression and T-wave changes: New horizontal or down-sloping ST depression ≥0.05 mV in two contiguous leads; and/or T inversion ≥0.1 mV in two contiguous leads; and/or T inversion ≥0.1mV in two contiguous leads with prominent R-wave or R/S ratio >1

– Evidence of ischemia on stress testing with cardiac imaging

– Evidence of ischemia on stress testing with angiographic evidence of ≥70% lesion and/or thrombus in an epicardial coronary artery or initiation/increased dosing of antianginal therapy

– Angiographic evidence of ≥70% lesion and/or thrombus in an epicardial coronary artery

**• Late coronary revascularization**

Late coronary revascularization was defined as planned percutaneous coronary intervention or coronary artery bypass grafting because of stable coronary artery disease (CAD) with a newly positive functional test for ischemia at more than 90 days after coronary CTA.

Patients who meet the clinical scenarios as follows were defines as stable CAD.

– patients with ‘stable’ anginal symptoms, and/or dyspnea

– asymptomatic subjects in whom CAD is detected by coronary CTA

**Supplemental Table I** Factors associated with adverse CT findings

Data are presented as mean ± standard deviation, number (%), or median [25^th^, 75^th^ percentile].

ACE-Is, angiotensin-converting enzyme inhibitors; ALT, alanine aminotransferase; ARBs, angiotensin receptor blockers; AST, aspartate aminotransferase; CCB, calcium channel blocker; CTA computed tomography angiography; eGFR, estimated glomerular filtration rate; HDL, high-density lipoprotein; LDL, low-density lipoprotein; MACE, major adverse cardiac events.

Adverse CTA findings are defined as the presence of obstructive and/or high-risk plaque.
